# A practical slice averaged image method for precise CT size specific dose estimates

**DOI:** 10.1038/s41598-025-27035-4

**Published:** 2025-12-01

**Authors:** Yutaka Dendo, Keisuke Abe, Shu Onodera, Shingo Kayano, Hideki Ota, Kei Takase

**Affiliations:** 1 Department of Radiological Technology, Tohoku University Hospital, 1-1 Seiryo-machi, Aoba-ku, Sendai, 980-8574 Japan; 2 Department of Diagnostic Radiology, Tohoku University Hospital, 1-1 Seiryo-machi, Aoba-ku, Sendai, 980-8574 Japan; 3 Department of Diagnostic Radiology, Tohoku University Graduate School of Medicine, 2-1 Seiryo-machi, Aoba-ku, Sendai, 980-8575 Japan

**Keywords:** Computed tomography, Radiation dose, Size-specific dose estimate, Water-equivalent diameter, Dose optimization, Radiography, Computed tomography, Whole body imaging

## Abstract

Computed tomography (CT) is a widely used diagnostic tool, but variations in patient anatomy make accurate radiation dose estimation challenging. While size-specific dose estimates (SSDE) can serve as a more effective tool for dose prediction than conventional indices like CTDI_vol_ and DLP, calculating the water-equivalent diameter (D_w_) for each slice is time-consuming and often impractical. To address this, we propose a novel method, the SSDE slice-averaged image (SSDE_SAI_), which calculates D_w_ from a single image generated by averaging CT values across all slices. This approach captures anatomical variability while reducing calculation effort. We retrospectively analyzed CT data from 282 adult patients in three scan regions: chest, abdomen–pelvis, and chest–abdomen–pelvis (CAP). SSDE_SAI_ was compared with SSDE_center_ and the reference mean SSDE using regression analysis and root mean square error (RMSE). SSDE_SAI_ showed stronger agreement with mean SSDE than SSDE_center_ across all regions, achieving R^2^ values up to 0.991 and lower RMSE. These results suggest that SSDE_SAI_ is a more advanced approach for dose prediction and may serve as a practical alternative for routine clinical use.

**Supplementary Information:**

The online version contains supplementary material available at 10.1038/s41598-025-27035-4.

## Introduction

Computed tomography (CT) is an essential imaging modality in modern medicine, and its demand is steadily increasing^1^. However, optimizing radiation dose remains a critical challenge, particularly for patients undergoing repeated examinations^2^. While the actual absorbed organ dose is the dosimetric quantity of primary interest, direct measurement is impossible in retrospective clinical studies. The next-best alternative, organ dose estimation via Monte Carlo (MC) simulations, also presents practical challenges for routine implementation, as the requisite specialized software is both costly and time-consuming to use, limiting its universal adoption^3^. Although the volume CT dose index (CTDI_vol_) and dose-length product (DLP) are primarily dose indicators of tube output, they are often used as metrics for dose management due to their simplicity and universal availability on scanner dose reports. However, these indices are measured using standard phantoms and do not account for variations in individual patient anatomy.

The American Association of Physicists in Medicine (AAPM) Report 204 proposed SSDE as a method to personalize CT dose assessments by applying size-specific conversion factors to CTDI_vol_^4^. Initially, these conversion factors were based on geometric dimensions such as anteroposterior and lateral diameters. Subsequently, AAPM Report 220 introduced the water-equivalent diameter (D_w_), which accounts for X-ray attenuation differences between tissues, thereby improving the accuracy of SSDE estimation^5^. Furthermore, SSDE has proven useful for estimating organ and effective doses^6–8^.

The most accurate method for calculating SSDE is to determine it for each axial slice and derive the average over the entire scan range (mean SSDE). This approach serves as a unique dose index specific to the scan, as it considers patient-specific size variations, X-ray attenuation properties, and the effects of automatic tube current modulation (ATCM) across the scan range. However, mean SSDE calculation requires determining both D_w_ and modulated absorbed dose for each slice, which involves extensive calculations or specialized software, making it impractical for routine clinical use^9^. Consequently, alternative methods that balance accuracy and calculation efficiency have been explored.

One such alternative is SSDE_center_, which estimates SSDE using the D_w_ of a single representative slice (typically a central slice) and the mean CTDI_vol_ of the scan^9–11^. This method significantly reduces calculation efforts but fails to account for body size variations within the scan range, potentially leading to dose over- or underestimation in certain regions^12^.

To address these limitations, we propose a novel method for calculating SSDE using a slice averaged image (SSDE_SAI_). This approach generates a single representative image by averaging all axial slices and calculates D_w_ from this image. By maintaining calculation efficiency while effectively accounting for patient size variations across the entire scan range, SSDE_SAI_ integrates information from the full scan and provides a representative index for accurate dose estimation, compared to SSDE_center_, which relies on a single slice.

This study aims to validate the effectiveness of SSDE_SAI_ by comparing it with mean SSDE and SSDE_center_. The evaluation focuses on its accuracy and clinical feasibility. We propose that SSDE_SAI_ provides a reliable alternative to mean SSDE by demonstrating a high correlation and improved accuracy, while significantly reducing calculation efforts.

## Materials and methods

### Study design and data collection

#### Patient selection

This retrospective observational study was approved by the Ethics Committee of Tohoku University Graduate School of Medicine (Approval No. 2022-1-899). As the study involved no direct interventions, the requirement for written informed consent was waived by the Ethics Committee of Tohoku University Graduate School of Medicine. All methods were carried out in accordance with relevant guidelines and regulations.

The study included 120 patients who underwent chest-abdomen-pelvis (CAP) CT between July 1 and July 31, 2022, 120 who underwent chest CT during the same period, and 120 who underwent abdomen-pelvis CT between February 1 and July 31, 2022. All scans were performed without contrast enhancement.

We excluded patients with metallic implants and those unable to raise their arms. These conditions could increase tube output and, more critically for this study, introduce severe beam-hardening artifacts (e.g. dark bands) that alter local CT values. While methods that average across the entire scan range (i.e. meanSSDE and SSDE_SAI_) mitigate the impact of such artifacts, the SSDE_center_ method is highly susceptible to this bias if an artifact is present on the single central slice. Therefore, we excluded these cases to ensure a fair and unbiased comparison of the methods’ intrinsic performance.

Additionally, to focus the analysis on a standard adult population based on World Health Organization (WHO) classifications^13^, we included only patients with a body mass index (BMI) between 18.5 and 40.0 kg/m^2^. This criterion excludes patients at the extremes of the body size spectrum, for whom the scanner’s Automatic Exposure Control (AEC) may operate at its fixed minimum or maximum tube current limits rather than modulating dynamically. Consequently, a total of 98 CAP, 95 chest, and 89 abdomen-pelvis CT cases were analyzed.

For each patient, we recorded sex, age, weight, height, and radiation dose indices (CTDI_vol_ and DLP). The minimum required sample size for univariate linear regression analysis by scan region was calculated using G*Power version 3.1.9.6^14^, assuming an effect size (f2) of 0.35 and a significance level (α) of 0.05, resulting in a required sample size of 40 cases. Based on this estimation, the study period was established to ensure a sufficient number of cases.

**Fig. 1:**
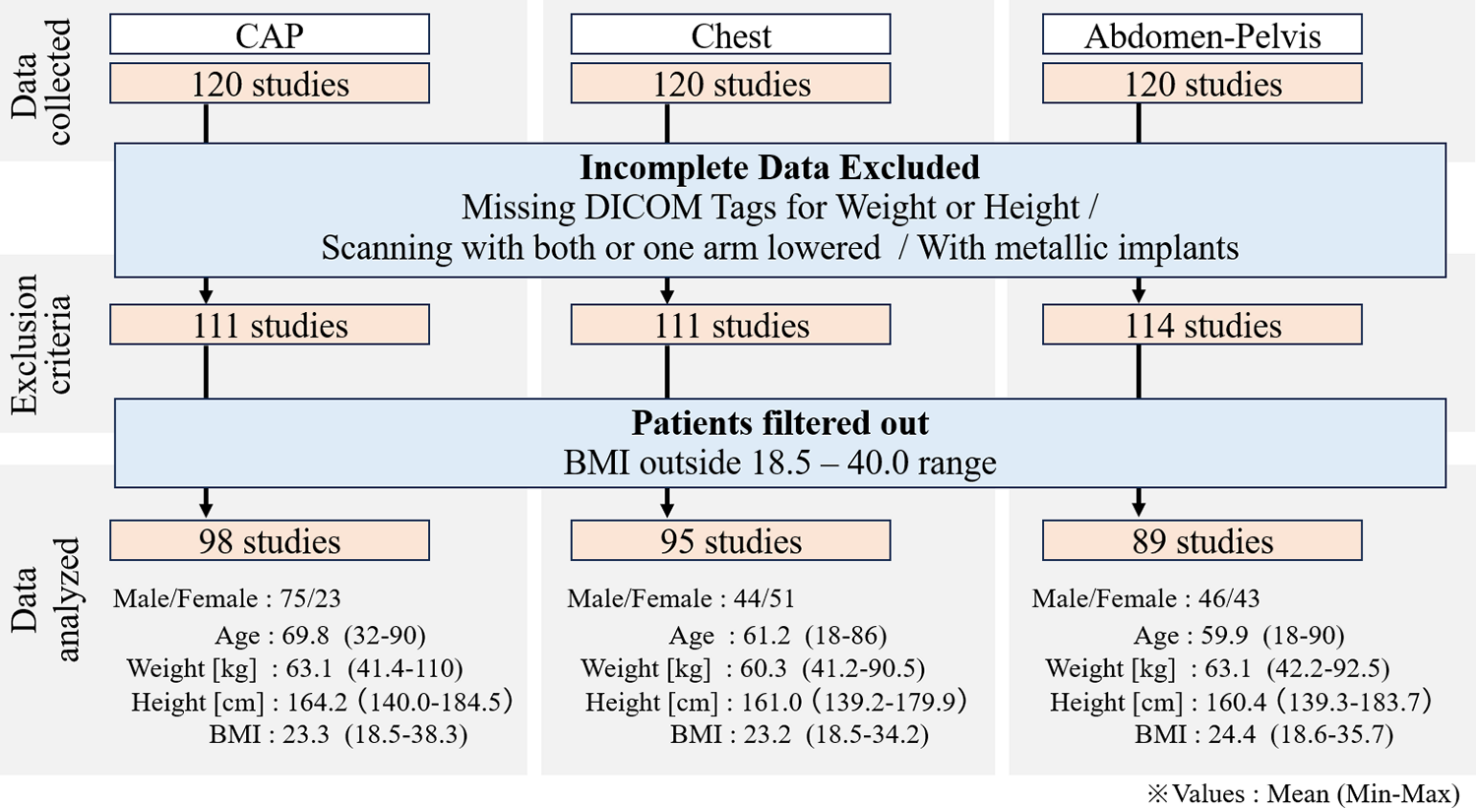
Patient selection flowchart.

The patient selection process is illustrated in Fig. 1.

### CT systems and image acquisition

Four CT scanners were used: the SOMATOM Definition Flash and SOMATOM Definition Edge (Siemens Healthineers, Erlangen, Germany), and the Aquilion ONE Vision Edition and Aquilion Precision (Canon Medical Systems, Tochigi, Japan).

All scans were conducted with patients in the supine position with their arms raised. The scan range and breath-holding conditions were determined based on specific examination types. Chest CT covered the region from the lung apex to the lower diaphragm during deep inspiration breath-holding. Abdomen-pelvis CT spanned from the top of the liver to the ischium and was performed during end-expiration breath-holding. CAP CT extended from the lung apex to the ischium and was performed during deep inspiration breath-holding. Helical scan mode was used for all acquisitions, with the tube voltage set to 120 kVp. ATCM was applied to optimize radiation dose, with CARE Dose4D (Siemens Healthineers) and VolumeEC (Canon Medical Systems) adjusting the tube current along both the z-axis and rotational direction. The gantry rotation speed was set to 0.5 s per rotation. The acquired slice thickness ranged from 0.5 to 0.6 mm, and the images were reconstructed using a soft tissue kernel to a 5 mm thickness. The field of view (FOV) was adjusted for each patient to ensure optimal clinical evaluation.

### Mean SSDE

#### *Calculation of CTDI*_*vol, z*_

Since ATCM was applied in this study, the volume CT dose index (CTDI_vol, z_) varied across slices within the scan range. Previous studies have reported that CTDI_vol, z_ values may not always be accurately stored in the digital imaging and communications in medicine (DICOM) tags of images^15^. Therefore, these values were recalculated using the following procedure:

First, the normalized CTDI_vol_, representing the CTDI_vol_ per unit milliampere-second (mAs) for the entire scan, was calculated using Eq. (1):

1 $$\text{normalized }{\text{CTDI}}_{\text{vol}}=\frac{\text{mean }{\text{CTDI}}_{\text{vol}}}{\text{mean mAs}},$$

where mean mAs represent the average mAs across the entire scan, and both mean CTDI_vol_ and mean mAs were obtained from the Radiation Dose Structured Report (RDSR).

Next, the CTDI_vol, z_ for each slice was calculated by multiplying the normalized CTDI_vol_ by the mAs(z) value obtained from the DICOM tags for each slice, as expressed in Eq. (2):

2 $${\text{CTDI}}_{\text{vol},\text{z}}=\text{normalized }{\text{CTDI}}_{\text{vol}}\times mAs\left(z\right).$$

### Calculation of SSDE

The SSDE for each slice along the z-axis (SSDE_z_) was calculated using CTDI_vol, z,_ and the conversion factor $f({D}_{w,z})$, as defined in Eq. (3):

3 $${\text{SSDE}}_{z}={\text{CTDI}}_{\text{vol},z}\times f\left({D}_{w,z}\right).$$

The conversion factor $f({D}_{w,z})$ was derived from AAPM Report 220^5^. Unlike the effective diameter, which considers only simple geometric indices such as lateral (LAT) and anteroposterior (AP) diameters, the D_w_ accounts for both geometric size and X-ray attenuation properties of the scanned body region, enabling more accurate dose estimation.

AAPM recommends calculating D_w_ with Eq. (4)^5^:

4 $${D}_{w}=2\sqrt{\left(\frac{1}{1000}\stackrel{-}{CT{(x,y)}_{ROI}}+1\right)\frac{{A}_{ROI}}{\pi }},$$

where $\stackrel{-}{CT{(x,y)}_{ROI}}$ represents the mean CT value within the region of interest (ROI), and A_ROI_ denotes the area of the ROI.

### ***Calculation of D***_***w, z***_*** and mean SSDE***

To improve the accuracy of SSDE calculations, a preprocessing step was implemented to remove the CT table from the scanned images and ensure precise D_w_ computation.

**Fig. 2:**
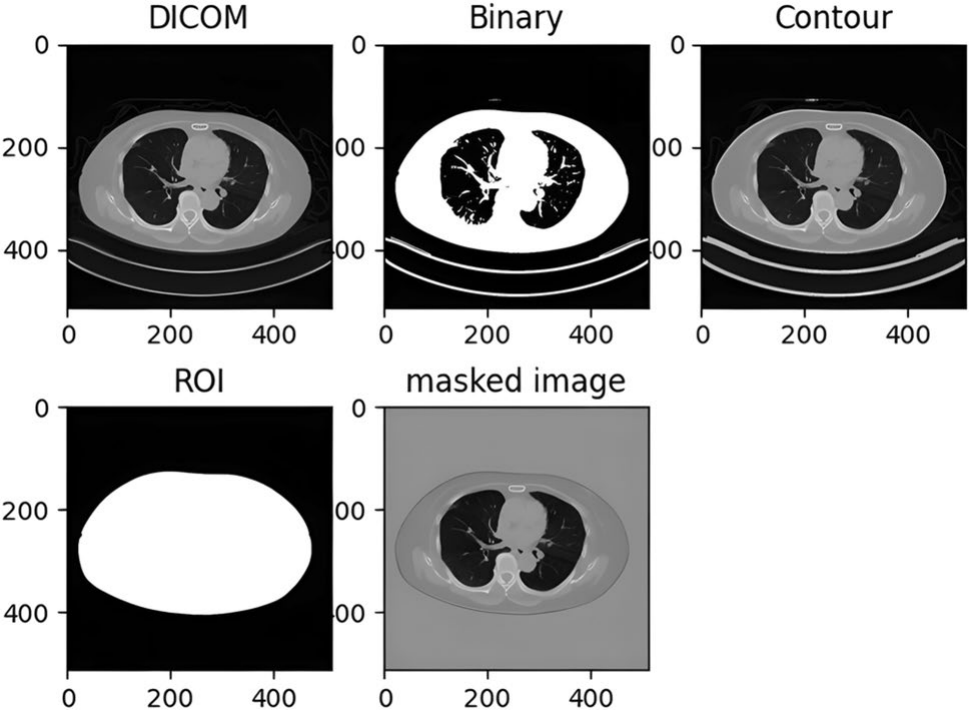
Image processing workflow for water-equivalent diameter computation.

First, the CT images were loaded, and a threshold of –900 Hounsfield Units (HU) was applied to extract image contours^9^. This contour extraction process segmented the patient’s body surface and CT table. Subsequently, the CT table was eliminated by retaining only the largest contour, leaving only the patient’s body surface. Finally, the ROI area (A_ROI_) and mean CT value within the ROI ($\stackrel{-}{CT{(x,y)}_{\text{ROI}}}$) were calculated. The entire process is illustrated in Fig. 2.

The water-equivalent diameter (D_w, z_) was then calculated using the AAPM-recommended Eq. (4)^5^. The SSDE conversion factor, $f({D}_{w,z})$, for each slice was determined using Eq. (5):

5 $$f\left({D}_{w,z}\right)=4.378094\times {e}^{-0.04331124\times {D}_{w,z}}.$$

The mean SSDE for the entire scan range, given N slices, was calculated using Eq. (6):

6 $$\text{mean SSDE}=\frac{{\sum }_{z=1}^{N}{\text{SSDE}}_{z}}{N}.$$

### ***SSDE***_***center***_

The SSDE_center_ was calculated using Eq. (7)^9^:

7 $${\text{SSDE}}_{\text{center}}={\text{mean CTDI}}_{\text{vol}}\times f\left({D}_{w,\text{center}}\right),$$

where $f({D}_{w,\text{center}})$ is determined by substituting the center slice values into Eqs. (4) and (5).

### ***SSDE***_***SAI***_

This study proposes SSDE_SAI_ as a novel dose evaluation index designed to accurately represent the dose distribution across the entire scan range while minimizing calculation efforts. This was achieved by computing the water-equivalent diameter (D_w, SAI_) using an integrated image obtained by averaging the CT values of all DICOM images within the scan range.

**Fig. 3:**
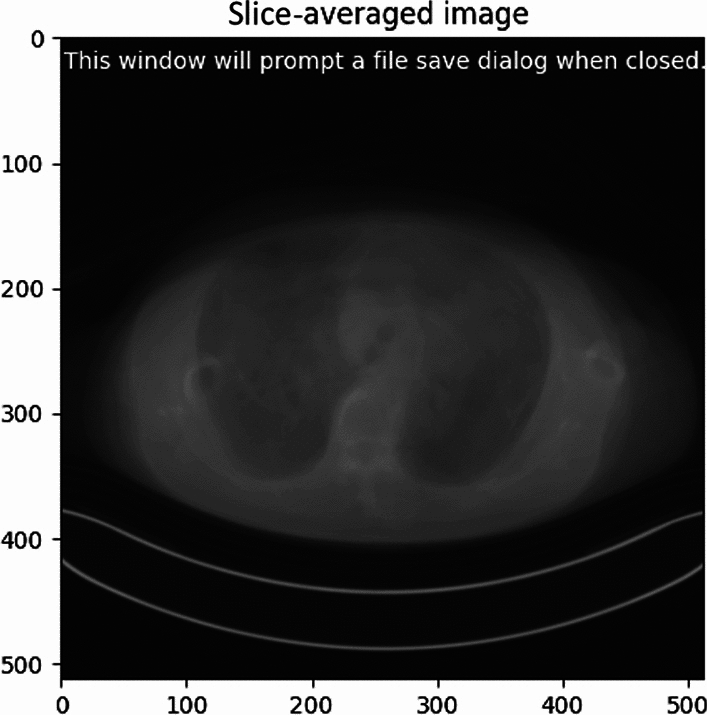
Slice averaged image generation program.

To generate the SAI, all DICOM images within the scan range were processed, and the CT values of each slice were averaged to produce a single integrated DICOM image. This procedure was implemented using a Python program specifically developed for this study, as illustrated in Fig. 3.

The integrated image underwent the CT table removal process described in Sect. “Calculation of D_w, z_ and mean SSDE”, which extracted only the patient’s body region. Subsequently, the conversion factor $f({D}_{w,SAI})$ was calculated using Eqs. (4) and (5), and SSDE_SAI_ was derived using Eq. (8):

8 $${\text{SSDE}}_{\text{SAI}}={\text{mean CTDI}}_{\text{vol}}\times f\left({D}_{w,SAI}\right).$$

These procedures were developed and implemented using Python (version 3.11.5, Python Software Foundation, Wilmington, DE, USA). PyDICOM, NumPy (version 1.26.4), and Matplotlib (version 3.8.0) were used for DICOM file processing, numerical analysis, and data visualization, respectively.

### Comparison metrics and statistical analysis

In this study, mean SSDE was selected as the reference standard for evaluating the accuracy of the dose estimation methods. While MC simulations are often considered the gold standard, different software packages can produce inconsistent dose estimates for the same scan due to factors such as varying phantom libraries or radiation transport models^3,16^, potentially introducing a software-specific bias. We therefore chose mean SSDE as the most appropriate reference. Mean SSDE is a dose index whose validity has been established; it is an unambiguously calculable metric that eliminates inter-software variability, accounts for longitudinal anatomical variation, and its correlation with MC-derived organ doses has been reported in several studies^3–6^. Using mean SSDE as our reference standard thus allowed for a robust evaluation of our method’s performance against a consistent and validated standard, free from the confounding bias of a specific MC software choice.

The accuracy of each method was evaluated against these reference standards. We performed linear regression analyses to assess the agreement between the estimated values (D_w, center_, D_w, SAI_) and the reference values (meanD_w_), and between the dose estimates (SSDE_center_, SSDE_SAI_) and meanSSDE. The agreement was quantified using the coefficient of determination (R^2^) and the root mean square error (RMSE).

The Shapiro-Wilk test was performed to assess the normality of the error distributions for each method relative to the reference values. Based on these results, an appropriate statistical test was selected to evaluate the significance of the differences. All statistical analyses were conducted using Python (version 3.11.5).

## Results

**Figure 4:**
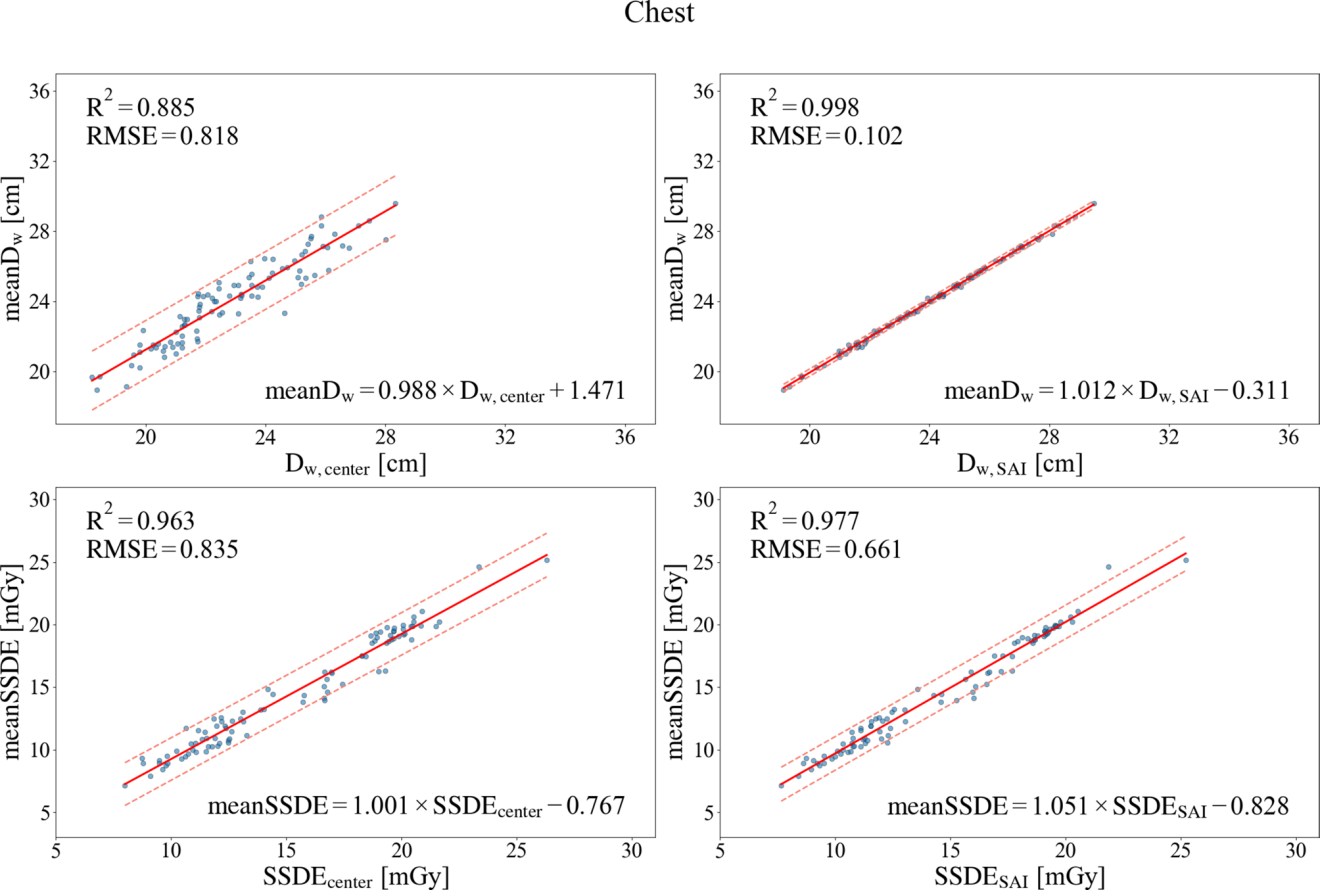
Regression analysis of D_w_ and SSDE in the chest region. The solid red line indicates the linear regression, and the dashed lines represent the 95% prediction interval.

**Figure 5:**
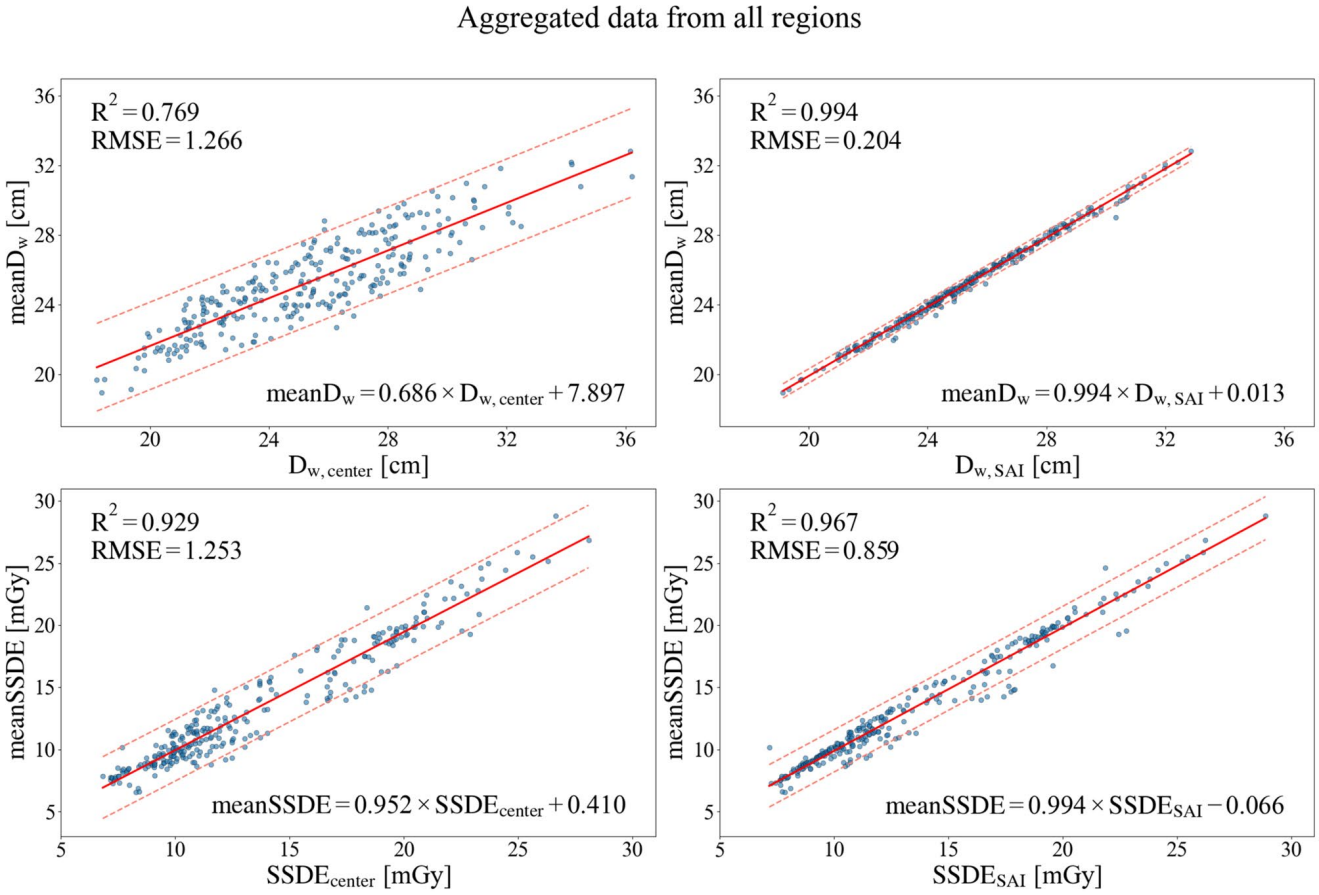
Regression analysis of D_w_ and SSDE for aggregated data from all anatomical regions. The solid red line indicates the linear regression, and the dashed lines represent the 95% prediction interval.

**Figure 6:**
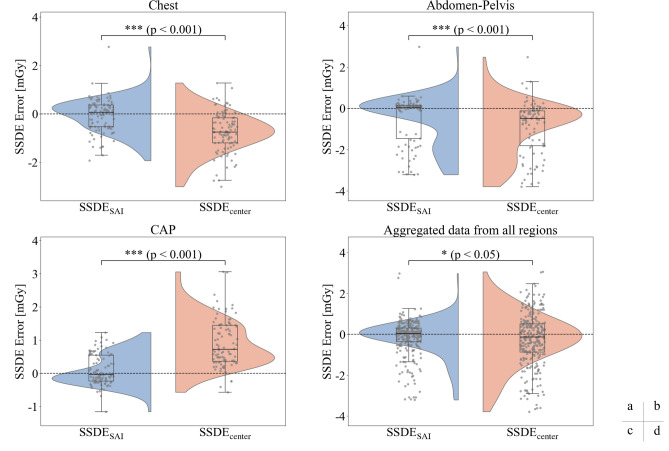
Half-violin plots showing the distribution of SSDE estimation errors, defined as meanSSDE − method-derived SSDE. The analysis is shown for (**a**) the chest, (**b**) abdomen–pelvis, (**c**) CAP regions, and (**d**) aggregated data.

In this study, we analyzed three anatomical regions: the chest, abdomen-pelvis, and CAP. To present our findings clearly, the main manuscript features the regression analyses for two key datasets: the chest region, as a representative case that demonstrated the most pronounced difference between the methods (Fig. 4), and the aggregated data, which illustrates the overall performance (Fig. 5). Regression plots for the other two regions (abdomen-pelvis and CAP), which exhibited similar trends, are available in the Supplementary Information (Supplementary Figs. S1 and S2, available online), while detailed quantitative results for all regions are provided in Tables 1 and 2 of the main manuscript. Additionally, to facilitate a direct comparison of estimation error distributions and reveal subtle differences among the groups, the half-violin plots for all three anatomical regions and the aggregated data are presented (Fig. 6).

**Table 1:** Regression analysis of D_w_.

Independent variable	D_w, center_				D_w, SAI_			
Regression	y = a*x + b (y: mean D_w_, x: D_w, center_ or D_w, SAI_)							
	a	b	R^2^	RMSE	a	b	R^2^	RMSE
Chest	0.988	1.471	0.885	0.818	1.012	–0.311	0.998	0.102
Abdomen-Pelvis	0.805	5.653	0.907	0.796	1.007	–0.495	0.993	0.222
CAP	0.743	5.158	0.904	0.728	1.023	–0.662	0.997	0.138
Aggregated data	0.686	7.897	0.769	1.266	0.994	0.013	0.994	0.204

**Table 2:** Regression analysis of SSDE.

Independent variable	SSDE_center_				SSDE_SAI_			
Regression	y = a*x + b (y: mean SSDE, x: SSDE_center_ or SSDE_SAI_)							
	a	b	R^2^	RMSE	a	b	R^2^	RMSE
Chest	1.001	–0.767	0.963	0.835	1.051	–0.828	0.977	0.661
Abdomen-Pelvis	0.928	0.02	0.933	1.235	0.941	0.237	0.943	1.140
CAP	1.044	0.373	0.98	0.675	0.998	0.134	0.991	0.458
Aggregated data	0.952	0.41	0.929	1.253	0.994	–0.066	0.967	0.859

### ***Regression analysis with mean D***_***w***_*** and mean SSDE***

Figures 4 and 5 and the online Supplementary Figs. S1 and S2 present the results of the regression analysis.

Across all anatomical regions, D_w, SAI_ and SSDE_SAI_ exhibited higher accuracy than D_w, center_ and SSDE_center_. Regarding D_w_, D_w, SAI_ in the chest region demonstrated excellent agreement with the mean D_w,_ with a coefficient of determination (R^2^) = 0.998 and RMSE of 0.102.

Similarly, in the regression analysis of SSDE, SSDE_SAI_ in the CAP region exhibited the highest R^2^ (0.991) and the lowest RMSE (0.458), highlighting the superior performance of SSDE_SAI_.

In the abdomen-pelvis region, the difference in accuracy between SSDE_SAI_ and SSDE_center_ was smaller compared to other regions. However, SSDE_SAI_ consistently outperformed SSDE_center_, reinforcing the validity of the SAI method as an effective approach for obtaining estimates closer to the mean SSDE.

When data from all anatomical regions were combined, D_w, SAI_ and SSDE_SAI_ showed strong agreement with mean D_w_ and mean SSDE, respectively. For SSDE_SAI_, R^2^ (0.967) and RMSE (0.859) were comparable to or even lower than those obtained from the regression equations of individual regions, such as the chest and abdomen-pelvis regions. Tables 1 and 2 provide the detailed numerical results.

### Comparison between methods

Figure 6 shows the distribution of SSDE estimation errors for each method.

In this study, we used half-violin plots to visualize the distribution of SSDE estimation errors (Fig. 6). We defined the error as the difference between the reference meanSSDE and the method-derived SSDE (mean SSDE − method). Consequently, a positive error indicates that the method underestimated the dose, while a negative error indicates an overestimation. Half-violin plots were chosen as they intuitively illustrate the shape of the error distribution and are well-suited for visually comparing differences in medians and variances.

The results of the normality test indicated that none of the error distributions followed a normal distribution in any anatomical region. Therefore, the Wilcoxon signed-rank test, appropriate for non-normally distributed data, was employed to compare the differences between methods. Consequently, SSDE_SAI_ and D_w, SAI_ exhibited statistically significant differences (p < 0.001) compared to SSDE_center_ and D_w, center_ across all anatomical regions.

An examination of the distribution shapes revealed that the error distribution of the SAI method was concentrated near zero relative to mean D_w_ and mean SSDE, while the center slice method exhibited a larger variance in errors, as shown in the half-violin plots. These findings demonstrate that the SAI method offers a more consistent dose index compared to the center slice method.

Furthermore, in the regression equations derived from the plots across all anatomical regions, the error distributions were centered around zero. However, the variance differed significantly between SSDE_center_ and SSDE_SAI_, with SSDE_SAI_ demonstrating a substantially narrower distribution, further reinforcing its higher consistency. This trend was observed across all anatomical regions, with SSDE_SAI_ exhibiting the narrowest distribution in each corresponding panel of Fig. 6.

## Discussion

This study evaluated the accuracy of the SAI and center slice methods for estimating SSDE and D_w_. The SAI method was proposed as a more effective approach for dose assessment by accounting for patient body size variations. The effectiveness of both methods was assessed by comparing their estimates to reference values (mean SSDE and mean D_w_) in the chest, abdomen-pelvis, and chest-abdomen-pelvis (CAP) regions. The findings revealed that SAI significantly outperformed the center slice method across all regions, with the most pronounced improvement in SSDE accuracy observed in the CAP region. These results were further compared to those of previous studies, and their clinical applicability is discussed.

While numerous studies have investigated the utility of the center slice method, only a few have specifically evaluated its accuracy in estimating mean D_w_ from the D_w, center_. Sarmento et al.^17^ conducted regression analyses of mean D_w_ derived from the center slice method for the chest and CAP regions, reporting R^2^ of 0.917 and 0.888, respectively. Although the accuracy trends for the chest and CAP regions differed from those observed in this study, the overall error magnitudes were comparable.

Particular attention to the coefficients and intercepts of the regression equations derived for D_w, center_ in this study is warranted. In the linear regression, these parameters are optimized to best fit the dataset, which may result in deviations from the ideal $y=x$ relationship. Such deviations may stem from limitations in sample size or imbalances in the sex distribution within the study population. Therefore, careful consideration is necessary when generalizing these regression equations.

Conversely, the regression equation derived in this study to estimate mean D_w_ from D_w, SAI_ demonstrated an exceptionally high R^2^ and low RMSE, despite the same sample size. Furthermore, this regression equation closely adhered to the $y=x$ relationship, requiring minimal adjustments to its coefficients and intercepts. These findings underscore the utility of D_w, SAI_, derived from the SAI, as a reliable and accurate indicator for estimating mean D_w_.

Among the indicators that accurately reflected patient size, mean D_w_, calculated using all slices, was regarded as the most reliable reference^9^. However, this approach involves extensive manual calculations, posing challenges to its rapid implementation in clinical settings. To address this limitation, an *n*-slice method (D_w, n_), which utilizes a subset of slices, has been proposed. For example, Anam et al.^12^ demonstrated that a nine-slice method can estimate mean D_w_ with less than 1% error, substantially reducing calculation efforts while maintaining practical accuracy.

The D_w, SAI_ method proposed in this study maintains the high accuracy of mean D_w_ by incorporating all slices while enabling a single computation that comprehensively utilizes patient-specific information. Unlike conventional *n*-slice methods, which involve selecting specific slices and performing repeated calculations, D_w, SAI_ is derived from the integration of all slices, eliminating the need for additional computations or slice selection and ensuring consistency of results. Furthermore, its strong agreement with mean D_w_, along with regression coefficients close to 1 and intercepts near zero, suggests its potential applicability across diverse patient populations.

Although the effectiveness of D_w, SAI_ in estimating D_w_ was clearly demonstrated, the impact of SSDE_SAI_ on SSDE estimation was relatively limited. The ideal method for calculating the mean SSDE is given by:

9 $$\text{mean} \text{SSDE}=\frac{1}{N}\left\{{\sum }_{z=1}^{N}\left[f({D}_{w,z})\times {\text{CTDI}}_{\text{vol},z}\right]\right\}.$$

In contrast, SSDE_SAI_ is calculated as follows:

10 $${\text{SSDE}}_{SAI}=f\left(\frac{{\sum }_{z=1}^{N}{D}_{w,z}}{N}\right)\times \frac{{\sum_{z=1}^{N}\text{CTDI}}_{\text{vol},z}}{N}.$$

Since these equations are not mathematically equivalent, SSDE_SAI_ does not perfectly match the mean SSDE. However, our results confirmed that SSDE_SAI_ achieved higher accuracy than SSDE_center_ while substantially reducing calculation efforts. Notably, its ability to incorporate information from all slices, unlike SSDE_center_, offers a significant advantage for clinical applications.

A deeper analysis of the error distributions in Fig. 6 provides insight into the conditions under which SSDE_SAI_ may be a less accurate surrogate for meanSSDE. While the error distribution for the chest was relatively symmetric, the abdomen-pelvis and CAP regions exhibited subtle asymmetric distributions. We hypothesized that this might be because the inherent mathematical non-equivalence between the SSDE_SAI_ and meanSSDE calculations is exacerbated by the significant anatomical heterogeneity present in these regions. To investigate whether this variability was linked to simple patient demographics, we performed a correlation analysis between the estimation error and patient characteristics (age, sex, height, weight and BMI). The results are presented in Supplementary Fig. S3, which is available online.

The results confirmed that the SSDE_SAI_ error had no strong correlation with any demographic parameter in any region, reinforcing the overall robustness of our method. In contrast, the SSDE_center_ error showed a notable correlation with weight and BMI in the heterogeneous CAP region (r ≈ 0.3), highlighting the inconsistency of the conventional method. Interestingly, the cause of the persistent asymmetry in the SSDE_SAI_ error was not explained by these demographic data. This leads us to conclude that these systematic errors are likely attributable to more complex anatomical factors not captured by BMI alone, such as visceral fat content, waist definition, or pelvic bone morphology, and/or even differences in CT scanner characteristics. While a retrospective review of individual patient images to confirm this hypothesis was beyond the scope of this study, our findings clarify the existence of specific conditions under which the SAI method’s accuracy may be slightly reduced. This represents an important consideration for its clinical application and a direction for future research.

Additionally, the error distribution of SSDE_SAI_ relative to the mean SSDE, as derived from regression equations using all-region aggregated data, exhibited a notably narrow shape. Previous studies on the center slice method have introduced region- and sex-specific regression equations to improve SSDE estimation accuracy^10,11^. However, in clinical practice, scan regions and ranges can vary. For example, a chest CT scan may extend into the upper abdomen, or an abdomen-pelvis CT scan may include portions of the lung fields. Under such conditions, applying different regression equations to each anatomical region may paradoxically reduce estimation accuracy.

The most pronounced improvement in SSDE accuracy was observed in the CAP region. This enhancement may be attributed to the longer anatomical coverage and greater variability in patient body size along the z-axis. In such extensive scan ranges, relying on a single representative slice—as in the center slice method—tends to oversimplify anatomical complexity. In contrast, SSDE_SAI_ integrates information across the entire scanned volume, providing a more comprehensive and stable estimation of patient size and corresponding radiation dose.

The regression equation derived in this study demonstrated its applicability across various scan regions, with a regression coefficient (*a*) close to 1 (0.994) and an intercept (*b*) near zero (–0.066). These findings suggest that SSDE_SAI_ can serve as a reliable dose index that closely approximates mean SSDE in trunk CT scans, without requiring additional corrections.

Currently, although standard CT systems promptly display mean CTDI_vol_ on the console post-imaging, they typically lack automated functions for calculating and displaying mean SSDE or mean D_w_. As a result, determining D_w_ across all slices necessitates dedicated dose calculation software, which poses challenges for real-time clinical implementation. To address this limitation, the center slice method, which estimates size-specific parameters using a single slice, has been traditionally employed.

The SAI method proposed in this study offers a practical alternative to the reference meanSSDE. Calculating meanSSDE is computationally intensive and time-consuming in a clinical setting, as it requires deriving D_w_ from every slice in the scan range, which can often number in the hundreds. This process can take tens of minutes per patient, making it impractical for routine workflow. In contrast, the D_w, SAI_ method addresses these computational challenges. Many CT systems are equipped with built-in image-averaging functions and clinical tools that enable manual ROI selection and the measurement of mean CT values and areas. By utilizing these existing features, D_w, SAI_ can be computed with minimal effort in under a minute, eliminating the need for specialized software, as illustrated in Supplementary Fig. S4.

The AAPM Report 246^3^ describes the center slice method as follows: “To estimate a single SSDE value for a given patient, the ‘shortcut’ of using the scanner-reported mean CTDI_vol_ and D_w_ derived from a central image within the scan range is considered acceptable.” This study demonstrated that the SAI method achieves superior accuracy compared to the conventional center slice method in estimating both D_w_ and SSDE. Notably, SSDE_SAI_ provides values that closely approximate the mean SSDE while minimizing calculation efforts, underscoring its potential utility in clinical practice. However, further validation studies are required to expand the applicability of the SAI method.

This study has several limitations. The relatively small sample size necessitates caution when generalizing the derived regression equations. Additionally, the study focused exclusively on adult patients, so its applicability to pediatric populations remains to be validated. Moreover, all scans were performed at a nominal 120 kVp, and a sub-analysis for each of the four scanner models was not conducted. Therefore, the influence of different nominal tube voltages or specific scanner characteristics (e.g. AEC implementation and differences in effective tube voltage) on the performance of the SAI method was beyond the scope of this work and remains a topic for future investigation. Despite these limitations, the study’s primary objective of comparing the center slice and SAI methods was successfully achieved.

## Conclusion

This study proposed and validated a novel method, the SAI approach, to improve the accuracy of SSDE and D_w_ evaluations.

Compared to the conventional center slice method, the SAI method exhibited stronger agreement with mean SSDE and mean D_w_, with a particularly notable improvement in accuracy observed in the CAP region. Furthermore, the SAI method significantly reduced calculation effort while integrating information from all slices, demonstrating its practicality for dose assessment.

This method provides a consistent dose index that contributes to high-accuracy dose evaluations without requiring additional corrections, making it suitable for seamless incorporation into existing clinical workflows. Future research should explore the applicability of the SAI method by validating its effectiveness across diverse patient populations and CT examination protocols.

## Supplementary Information

Supplementary Information.

## Data Availability

An anonymized version of the dataset used in this study is available in Supplementary Table S1, in accordance with ethical considerations.
