# Supplementary material for: A practical slice averaged image method for precise CT size specific dose estimates
Source: Sci Rep. 2025 Dec 1;15:42870. doi: 10.1038/s41598-025-27035-4 (PMC12669235; doi:10.1038/s41598-025-27035-4)
Supplement: Supplementary file 1 — Supplementary Information. [file 41598_2025_27035_MOESM1_ESM.pdf]

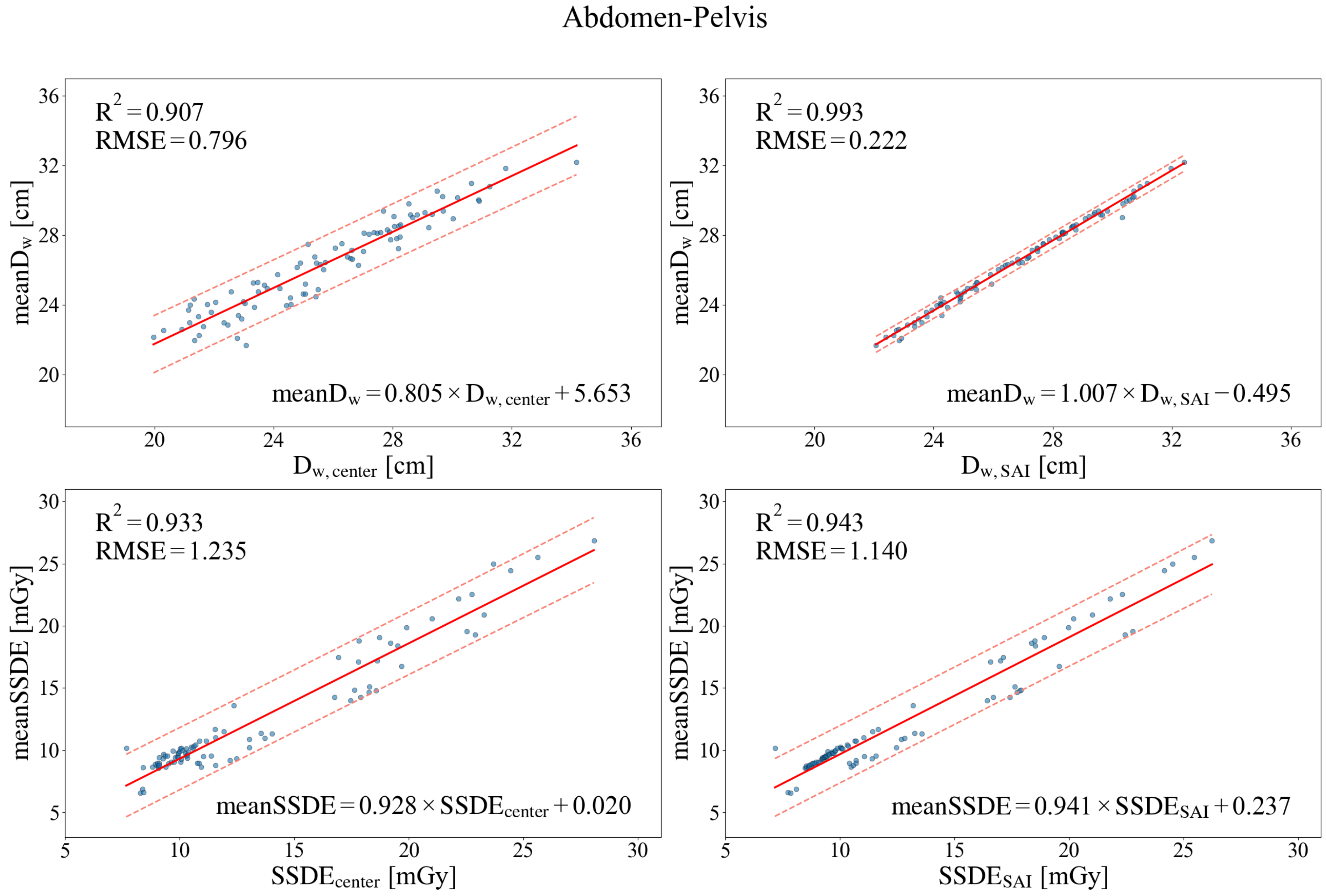

Supplementary Figure S1: Regression analysis of D_w and SSDE in the abdomen-pelvis region. The solid red line indicates the linear regression, and the dashed lines represent the 95% prediction interval.

CAP

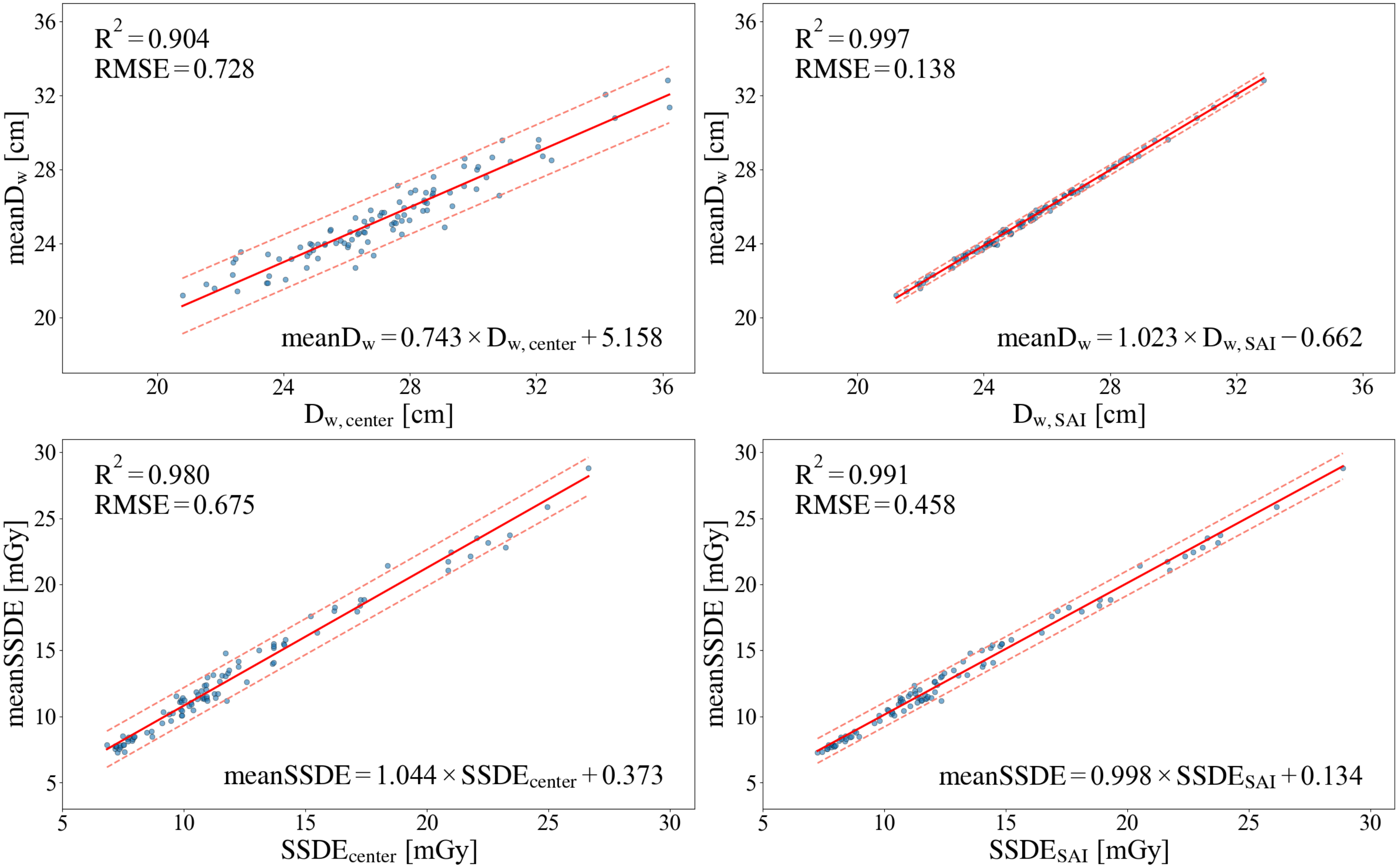

Supplementary Figure S2: Regression analysis of D_w and SSDE in the CAP region. The solid red line indicates the linear regression, and the dashed lines represent the 95% prediction interval.

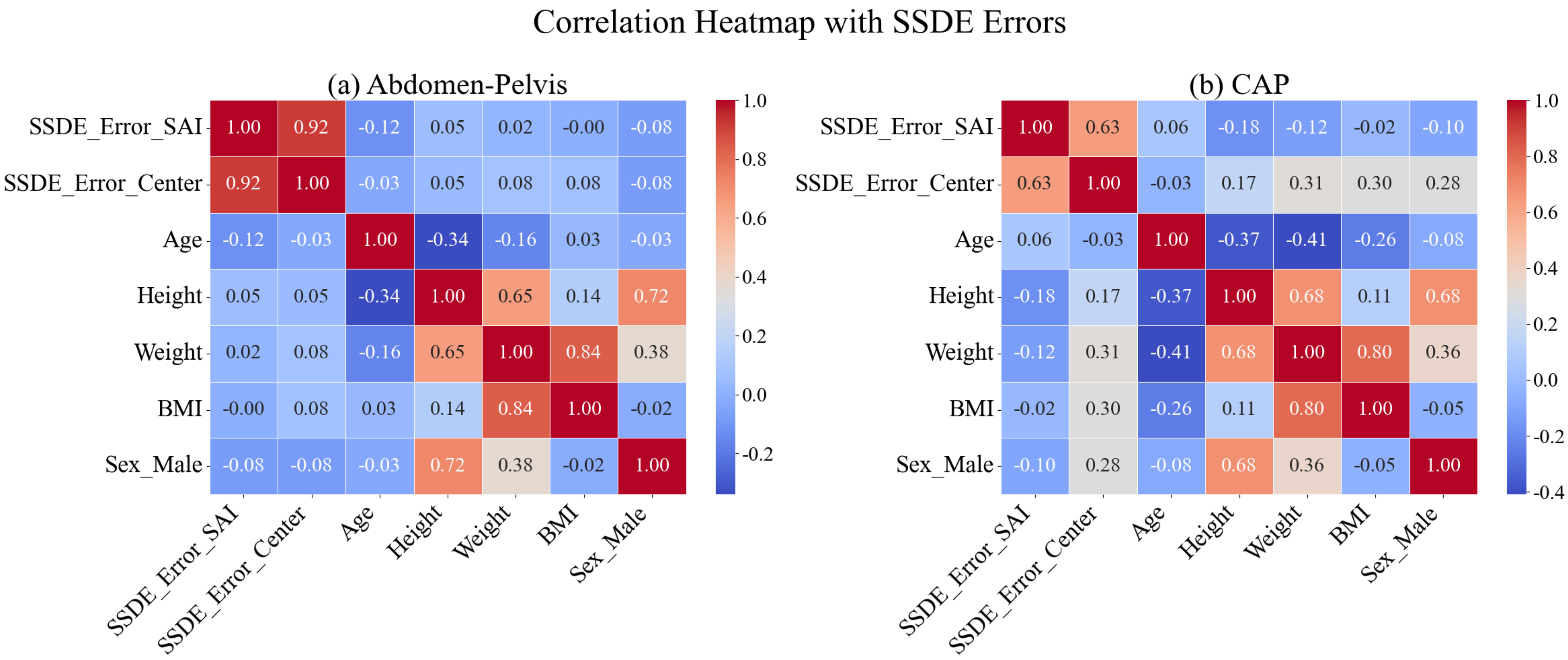

Supplementary Figure S3: Correlation heatmaps for SSDE errors. (a) Abdomen-Pelvis and (b) CAP heatmaps show the correlation between SSDE errors ($SSDE_{SAI}$ Error and $SSDE_{center}$ Error) and various patient characteristics.

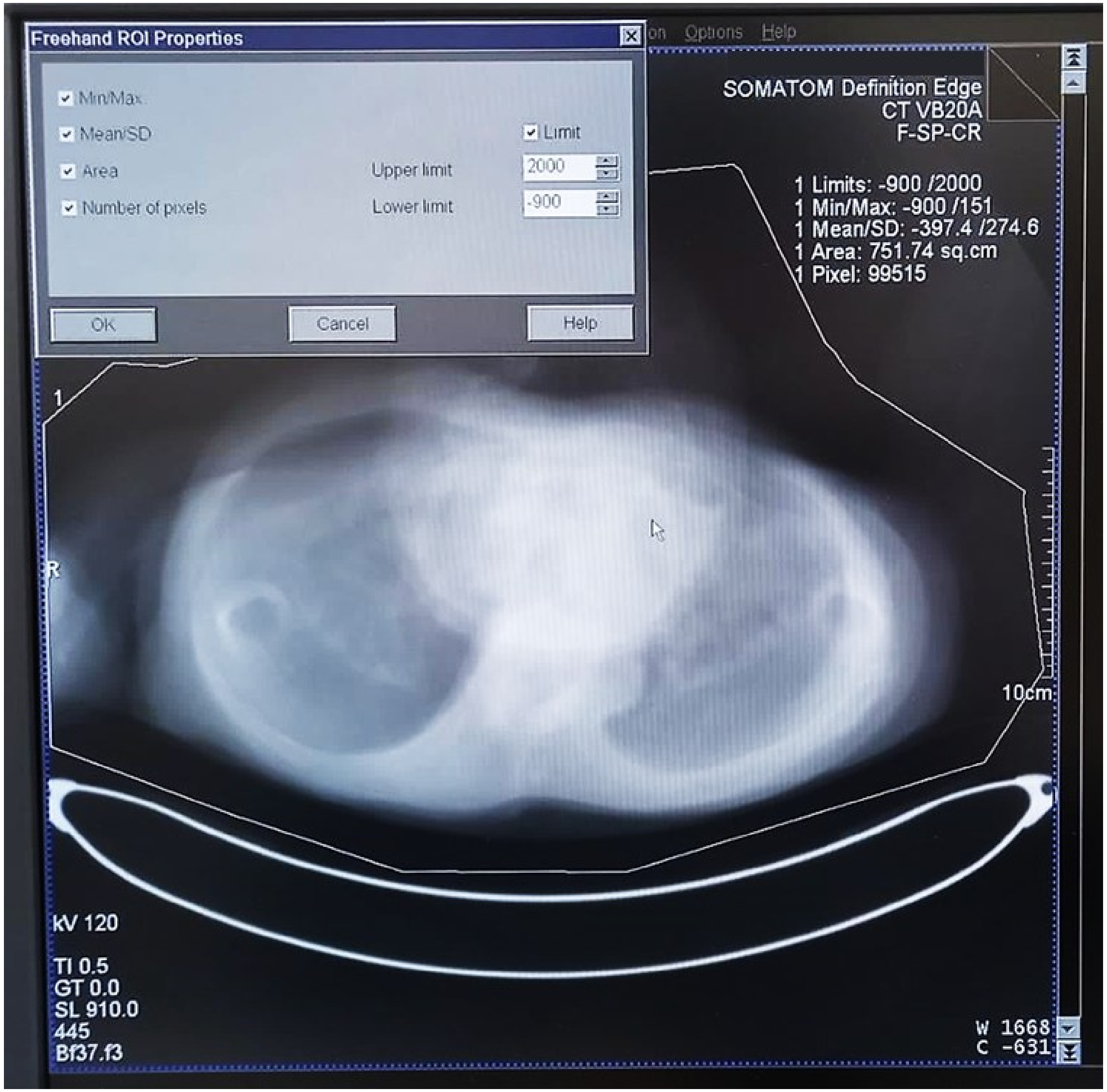

Supplementary Figure S4: Practical Implementation of the $D_{w,SAI}$ Method Using Standard CT System Functions

Supplementary Table S1: Anonymized subject data and corresponding dose metrics (Dw and SSDE).
ID prefixes indicate scan regions: A = Abdomen–Pelvis, C = Chest, T = Trunk (CAP).

ID	SEX	Age	Height	Weight	BMI	meanSSDE	meanDw	SSDE_SAI	Dw_SAI	SSDE_center	Dw_center
A003	F	77	153.9	84.5	35.7	10.11006425	30.99217717	10.08584427	31.16369735	10.28599357	30.62855095
A004	F	40	158.5	88.1	35.1	25.50487242	31.85765026	25.45662603	31.97167049	25.62915063	31.7877257
A006	F	55	161.0	86.1	33.2	11.67043605	30.78231078	11.67485139	30.92313766	11.53659329	31.24757311
A007	M	48	168.3	91.8	32.4	14.78497334	30.22678118	17.86447262	30.72112011	18.57454622	29.65960434
A008	M	87	170.5	92.5	31.8	13.58120252	32.20927968	13.19817914	32.40462011	12.36850867	34.17276748
A009	F	66	152.0	71.0	30.7	14.81348466	29.98393583	17.90012195	30.48343664	17.62843363	30.89995741
A010	F	42	157.1	75.4	30.6	22.55057821	28.59029772	22.32710472	28.77847595	22.75198237	28.26509895
A011	F	44	161.0	77.1	29.7	11.48405738	29.068368	11.42132511	29.20436199	11.92114568	28.03790571
A012	M	50	174.9	90.6	29.6	11.01262896	30.55819582	11.05314386	30.72419466	11.56744547	29.48561601
A013	F	69	150.4	66.4	29.4	19.8777394	27.90293814	19.9940512	28.1072	19.89363661	28.24431774
A014	M	68	167.8	80.5	28.6	14.64596435	29.16935677	17.73287516	29.61201752	18.26392529	28.80842127
A015	M	46	170.0	82.6	28.6	19.27555917	30.17372538	22.45850947	30.67500543	22.88467093	30.16307641
A016	F	66	159.4	72.5	28.5	19.57164748	30.04675525	22.78243816	30.57473134	22.52961526	30.87863922
A017	F	40	168.5	79.7	28.1	10.1463528	29.80006372	7.171804633	30.34659826	7.667581544	28.52619837
A018	M	45	173.6	84.3	28	24.46797626	29.31000326	24.15816906	29.39265182	24.44356158	29.07281342
A019	M	69	168.6	79.5	28	14.00288321	29.00282463	16.4219727	30.32683791	17.45475497	28.6658109
A020	M	70	165.4	76.4	27.9	22.16204285	28.18411152	21.79989187	28.33977164	22.16863277	27.88297346
A021	M	52	175.0	85.2	27.8	26.83585487	29.39607965	26.24791656	29.52297285	28.08059047	27.68492414
A022	F	31	161.5	72.1	27.6	10.34005678	27.13487784	10.36088956	27.2948911	10.61866327	26.62562469
A023	M	71	167.0	76.6	27.5	24.98111628	28.9495536	24.53082192	29.07492628	23.69136165	30.02319609
A024	M	78	164.0	73.0	27.1	8.793074263	27.27796512	8.645608504	27.47213309	9.10526497	26.06139784
A025	M	51	172.0	80.2	27.1	10.73366902	28.52967608	10.67029199	28.68987378	10.86333546	28.20157649
A026	M	74	162.2	71.2	27.1	9.963732513	28.13446633	9.814325621	28.33852645	10.13108506	27.47344325
A027	F	72	155.0	65.0	27.1	10.73315591	28.06439509	10.7012751	28.31254839	11.15113211	27.19112054
A028	M	74	164.0	71.6	26.6	9.538436057	29.1981416	9.444541007	29.38446523	9.467036548	29.31967587
A029	M	84	162.1	69.9	26.6	9.660461532	28.46009573	9.496331572	28.61113565	9.291668542	29.20448545
A031	M	67	165.0	72.0	26.4	9.464921153	28.12368569	9.398105337	28.40696479	9.888041545	27.02300877
A032	M	59	168.7	74.9	26.3	10.14980875	27.51769239	10.06012905	27.66485268	10.58067327	26.29094381
A033	F	59	165.4	71.7	26.2	15.1104018	28.31180907	17.64441091	28.77199951	18.27942412	27.80910196
A034	M	41	166.5	72.5	26.2	16.76521159	29.3847346	19.57482935	29.82835353	19.67764331	29.68568742
A035	M	30	183.7	87.7	26	8.94793411	28.49602798	8.820916308	28.65644621	9.020678582	28.0465826
A036	F	81	143.0	53.0	25.9	18.63825458	26.41785155	18.37362384	26.6359622	19.20102727	25.43638737
A037	F	52	147.0	55.8	25.8	9.735923034	24.9604367	9.688765272	25.24906045	10.22433744	23.78378729
A038	F	73	151.4	58.9	25.7	9.321229654	27.73865491	9.273100142	27.87963789	9.261448173	27.91387934
A039	M	84	155.1	61.8	25.7	10.84968363	26.75331789	12.69797398	27.1814272	13.0355929	26.46678857
A040	M	72	157.1	62.8	25.4	10.96463476	26.76032307	12.834297	27.18188199	13.71726776	25.3699114
A041	F	82	156.6	62.4	25.4	9.741345343	26.03722181	9.738643273	26.15990488	9.914866944	25.67151065
A042	M	70	158.0	62.7	25.1	8.610286718	26.27319651	8.525920249	26.41005481	8.388495005	26.85259617
A043	F	83	145.0	52.3	24.9	11.35750413	26.6682156	13.26632544	27.13581187	13.53376447	26.59226409
A044	F	66	146.0	53.0	24.9	18.37347722	24.74927352	18.53418788	24.74927352	19.51866322	23.48267959
A045	M	18	166.8	67.6	24.3	8.65555873	26.63894227	8.744829537	26.81880235	8.802774576	26.63894227
A046	F	81	139.3	46.9	24.2	17.12402849	24.19428951	16.5886922	24.8921285	17.80103245	22.97120262
A047	M	55	174.3	72.8	24	9.03959891	27.50070161	9.14717253	27.66734798	10.03210051	25.15245776
A048	F	56	149.4	52.3	23.4	11.32350183	25.19288701	13.57311037	25.94581757	14.02240984	25.05892611
A049	M	61	166.6	64.9	23.4	9.40704706	27.24716211	9.327285365	27.49335179	9.095247641	28.17941952
A050	M	60	177.0	72.9	23.3	10.14570489	27.06111264	9.894211538	27.49335179	10.06997785	27.01380613
A051	F	71	150.6	52.6	23.2	9.448821768	25.14194907	9.333744695	25.38645487	9.935120297	23.68599842
A052	F	69	151.8	53.2	23.1	9.610303434	25.26884968	9.558599325	25.45211326	10.33979457	23.3126757
A053	M	62	160.0	59.0	23	9.397862903	26.1521791	9.265267093	26.30094358	9.796591136	24.78235014
A054	M	65	178.3	73.1	23	14.23170646	28.15432703	17.41467635	28.32193607	17.89428083	27.58205857
A055	F	86	147.8	50.2	23	9.498517632	24.89327322	11.07287473	25.4133658	11.04240745	25.48840294
A056	F	53	152.7	53.5	22.9	10.18060584	24.08323127	12.48225748	24.21988322	13.03647795	23.03676954
A057	F	42	149.0	50.6	22.8	20.55568954	29.17769042	20.19482844	29.66003017	20.99953024	28.5959184
A058	M	60	168.4	64.5	22.7	9.852390316	26.42197684	9.662850061	26.84427451	10.38135824	24.89099811
A059	M	17	166.8	63.0	22.6	8.893823214	25.75099419	8.91669045	25.89816804	9.513148982	24.13479103
A060	M	68	167.0	62.9	22.6	10.40602699	28.16629534	10.32261864	28.30940088	10.68883356	27.35998012
A061	F	45	149.2	50.1	22.5	8.97418438	22.84148594	8.87814144	23.11279561	9.094487664	22.45711331

A062	M	73	174.2	68.1	22.4	9.743287273	26.32307126	9.621941609	26.48822584	9.954631363	25.56250648
A063	M	18	167.5	62.7	22.3	9.047529432	25.28876841	9.066591277	25.44414289	9.751903292	23.45973777
A064	F	74	147.2	48.0	22.2	17.47004712	24.01498977	17.13018925	24.2600813	16.93538153	24.5715612
A065	F	71	148.0	48.5	22.1	9.947571985	24.62899608	9.786412504	24.81066047	9.697982137	25.05786264
A068	M	76	162.5	58.1	22	9.332295541	24.0101477	11.40352033	24.25133781	12.48806894	21.77712338
A069	F	66	161.5	57.0	21.9	9.535781729	24.62591867	9.378493555	24.91363533	9.355225684	24.9812853
A070	F	42	162.4	57.5	21.8	9.896090986	23.3916935	9.463446483	24.27744888	9.985638445	22.81469866
A071	F	75	152.0	50.0	21.6	9.354107232	23.98524568	9.236309372	24.23051244	10.32485123	21.19638241
A072	F	53	159.0	54.6	21.6	10.11927982	24.39629848	10.13407256	24.23051244	10.01052382	24.56456897
A073	F	45	154.7	51.5	21.5	20.90878577	23.71858093	21.01191063	23.93546889	23.28556462	21.13737691
A074	F	34	155.2	50.9	21.1	9.26355791	22.54305782	9.207525696	22.74568721	10.06850408	20.31125047
A075	M	87	146.9	45.6	21.1	8.783641758	22.98117639	10.59098899	23.59604759	11.57199265	21.18358225
A076	M	24	171.2	61.9	21.1	9.518374799	24.73989753	9.398187719	25.028328	10.2847167	22.57343559
A077	F	18	148.0	45.6	20.8	8.629516176	22.2461094	10.48074872	22.65221302	10.93328241	21.50101
A078	F	78	155.3	50.2	20.8	8.94416144	23.85250602	10.40912423	24.46533245	10.84178085	23.35625796
A079	F	64	161.0	53.9	20.8	8.87744951	23.21124047	8.754532994	23.49462719	8.941005412	22.9206407
A081	M	76	163.0	53.7	20.2	8.538427404	23.5651365	8.48883861	23.74403044	9.086312282	21.89168546
A082	M	70	153.9	47.8	20.2	6.851030317	24.95536525	8.099207822	25.12283759	8.378399954	24.19987233
A083	F	57	155.5	48.2	19.9	17.18300126	22.14500946	17.02313541	22.39956914	18.61578505	19.96389135
A084	M	34	169.8	57.0	19.8	9.054788574	22.60631905	8.971328	22.8284368	9.623707487	20.91675177
A085	M	78	162.4	52.0	19.7	8.731929106	22.74838542	8.532538019	23.35457308	9.084737746	21.64678297
A087	F	73	146.7	42.2	19.6	18.80499819	21.67410242	18.50128821	22.06631923	17.83290597	23.06837785
A088	F	63	150.0	44.0	19.6	8.751037484	22.97898217	8.681071797	23.33225745	9.004948085	22.33471273
A089	M	90	160.0	50.0	19.5	9.543518803	24.46650716	11.57758671	24.90689841	11.3610252	25.42113363
A090	M	55	168.0	55.0	19.5	19.07280542	23.96757924	18.93017213	24.09731275	18.70638459	24.42117916
A091	M	36	171.1	56.0	19.1	8.655424823	24.13663921	8.624147254	24.33748436	9.378182542	22.05476828
A092	M	50	161.0	49.5	19.1	14.25678708	27.81656109	16.69814088	28.20902261	16.75064176	28.12353141
A093	F	81	151.0	43.5	19.1	8.938772668	22.09802017	10.69652114	22.92255827	10.75657354	22.77009111
A094	F	67	157.0	47.0	19.1	6.598016447	23.32396835	7.735798299	23.77742759	8.419418955	21.47122971
A095	M	60	170.5	55.0	18.9	9.171248996	24.33927827	10.69489485	24.88679541	12.19310583	21.31635874
A096	M	46	168.6	53.2	18.7	10.23664082	26.43120943	10.03095495	26.97052462	10.50085618	25.7237443
A097	F	39	154.6	44.5	18.6	6.569018475	21.96607992	7.849226706	22.83081439	8.28758313	21.35084978
C001	F	72	145.0	72.0	34.2	14.43787867	27.51879273	14.61382746	27.62768011	14.40354349	28.02240134
C002	F	63	150.6	77.6	34.2	11.15439311	27.59569907	12.30003492	27.57594201	13.26991396	25.50898763
C003	F	30	152.0	76.0	32.9	13.23194892	28.83676223	12.55825099	28.85328694	14.0193322	25.85597654
C004	M	64	166.0	86.0	31.2	25.16278148	29.57655836	25.21828491	29.48480396	26.30127931	28.33967925
C005	M	76	159.3	78.6	31	14.8500196	28.31473873	13.59188609	28.2945201	14.19985324	27.10281595
C006	M	60	174.0	90.5	29.9	18.51188169	28.33295185	17.78108253	28.16136296	19.34700029	25.86278674
C007	M	79	160.7	73.7	28.5	18.90247013	27.26464845	18.35630638	27.29429812	19.66576742	25.41773428
C008	M	63	170.0	81.0	28	24.63064278	27.84934223	21.8637706	28.09612818	23.35373749	26.30072487
C009	F	42	158.4	70.0	27.9	12.57765512	25.73075645	11.91464974	25.68586574	12.1622009	25.12583048
C011	M	53	165.0	75.0	27.5	18.99443548	28.59819761	18.13474514	28.61542952	18.92675341	27.45128302
C012	M	63	169.0	78.0	27.3	18.68089731	27.72551293	17.9332063	27.72687946	19.4378106	25.53277662
C013	M	72	161.0	70.6	27.2	14.10127812	27.08877532	16.03315468	27.08224651	16.6445339	26.0630808
C014	M	51	175.0	83.0	27.1	17.49592161	26.84025657	17.28367165	26.8381336	18.27339324	25.32166259
C016	M	47	179.9	86.5	26.7	19.19409701	25.62685992	19.13162832	25.5556363	20.08903275	24.22578959
C017	M	47	170.8	77.3	26.5	7.891415163	26.4084885	8.405154915	26.43724955	9.109068437	24.24698183
C018	F	40	152.7	61.5	26.4	10.90297544	24.90299116	10.78241365	24.96577023	11.54904997	23.09518427
C019	F	48	167.6	73.0	26	9.781169342	24.41109974	10.55371477	24.48149775	11.06963449	23.18169823
C021	M	86	170.0	74.3	25.7	10.23896978	27.1263129	11.12217688	27.09474743	11.61455226	25.91504894
C022	F	37	148.6	56.6	25.6	10.49125685	24.29906896	10.7248697	24.3528559	11.05374996	23.53028139
C023	F	74	156.9	63.0	25.6	18.68860035	27.1639117	18.52373741	27.04702196	18.84802514	26.57437963
C024	F	41	164.5	68.4	25.3	19.42471663	25.49468552	19.26470312	25.36686211	19.07255427	25.63985742
C025	F	67	158.0	63.0	25.2	9.864413831	23.12030484	10.67327227	23.18429594	11.50587796	21.13863628
C026	F	71	153.0	58.7	25.1	19.84646042	24.18433119	19.70742814	24.12199931	20.41628777	23.15963599
C027	F	79	147.3	54.4	25.1	10.45810898	22.61264314	10.3350389	22.61125305	10.85313494	21.27914917
C028	M	83	166.7	69.1	24.9	18.79795702	26.30562898	18.68210884	26.20315773	19.55100235	24.96511262
C029	M	73	159.5	63.2	24.8	16.21787555	25.32886116	15.89773035	25.38440527	16.65986382	24.10916257
C030	M	70	176.0	76.7	24.8	11.42655386	26.66124301	11.94814338	26.70795376	12.62265733	25.2123535
C032	M	45	168.1	69.8	24.7	19.27845912	25.85999403	19.20497338	25.86096562	20.14341935	24.56169561
C033	F	62	151.8	56.8	24.6	19.11962494	24.9755515	18.81014638	24.96552915	18.66184727	25.18108945
C034	M	63	172.5	72.7	24.4	18.79754438	26.44084963	18.64937638	26.45645231	20.43142371	23.97107782

C035	F	41	160.0	62.0	24.2	10.52021017	24.9048313	11.30960679	24.80140597	12.10645114	22.94718189
C036	F	78	152.7	56.1	24.1	19.32653265	25.7659738	19.15751389	25.72435094	18.89149336	26.10516562
C037	F	75	164.0	64.7	24.1	11.71582843	25.33063545	12.39063619	25.30278709	12.37557191	25.33591726
C038	F	69	160.6	62.0	24	10.81860928	25.37262278	11.20519008	25.44586664	11.35593284	25.08193737
C039	F	82	158.4	59.9	23.9	20.60953972	21.14795346	20.23691099	20.97269783	20.5273014	20.58468568
C040	F	22	160.0	61.1	23.9	16.10408393	24.2488981	16.63580036	24.29802565	16.97599497	23.74672904
C041	M	75	157.2	58.8	23.8	19.84524927	24.43869092	19.54824851	24.34286191	21.50089053	21.74998893
C042	M	82	164.9	64.6	23.8	19.05631556	25.93490605	19.07630777	25.84003579	19.87654435	24.72092001
C043	M	74	172.5	70.5	23.7	10.3042095	26.29273773	10.8365382	26.35692476	12.03294608	23.50489316
C044	M	64	169.5	68.0	23.7	17.52474866	25.56508531	16.92306052	25.61112993	18.26898597	23.52700794
C045	F	61	157.0	58.0	23.5	15.61518485	25.36365097	15.65617493	25.29703728	16.76285576	23.43698172
C046	F	29	149.2	52.3	23.5	10.72289802	22.32404411	11.41127613	22.35389883	12.48056558	19.91456831
C047	F	59	161.4	61.2	23.5	18.53896341	27.03491661	18.58238943	26.96092803	18.70622448	26.78004254
C048	F	76	152.6	54.7	23.5	19.74426562	23.29263643	19.47779719	23.36462186	19.68045463	23.08273301
C049	M	69	167.0	64.5	23.1	16.2880535	24.71125466	17.68832507	24.7995083	19.29282753	22.43485513
C050	F	72	148.5	50.4	22.9	11.53882099	21.71663249	11.11455763	21.87849936	11.17590567	21.72859389
C051	M	77	172.3	67.6	22.8	9.661267948	24.91162906	10.23789372	24.87755405	10.75111545	23.54546133
C052	M	73	165.8	62.5	22.7	11.89645612	23.44819276	11.55445726	23.52768203	12.32821502	21.76242116
C053	M	79	163.3	60.5	22.7	17.4455175	24.81836427	17.68443133	25.05671693	18.46022344	23.88747938
C054	M	83	159.9	57.8	22.6	10.26555079	24.27133786	10.78507733	24.31555138	11.77503257	21.9239538
C055	F	69	155.0	53.8	22.4	19.45684942	23.24012178	19.08105829	23.25808575	19.65358152	22.4529669
C056	M	58	170.0	64.5	22.3	16.2588784	24.28385289	17.20044959	24.46095254	19.0148643	21.72981956
C059	M	48	164.6	60.0	22.1	13.01457607	23.43378948	12.46088103	23.59409368	13.11783649	22.194869
C060	M	65	168.1	62.5	22.1	9.433007755	23.99697687	10.00384398	24.00416709	10.63054296	22.34940417
C062	F	86	139.2	42.8	22.1	19.76107906	21.63137322	19.13989898	21.55132785	19.37613277	21.21725537
C063	M	71	167.6	61.5	21.9	13.93034193	24.37799932	15.27827663	24.44471695	16.671391	22.06825611
C064	F	52	162.0	57.2	21.8	12.49000493	22.97676421	12.2550862	23.00671857	13.0091733	21.3805007
C065	F	38	166.8	60.6	21.8	10.92725184	21.5763474	11.29510232	21.84313995	11.9029041	20.41574061
C067	F	75	147.7	47.4	21.7	20.22342308	21.34100872	19.79246526	21.34569446	20.53709287	20.33992186
C068	M	63	157.8	53.6	21.5	20.20573595	24.17057018	20.30125082	23.93960633	21.62824825	22.21523718
C069	M	71	173.3	64.5	21.5	14.3314917	25.084281	14.27475173	25.14058535	15.76000472	22.44492308
C070	F	54	158.7	54.0	21.4	12.46535279	21.84990829	11.76958961	21.89821515	11.85179898	21.70865266
C071	M	46	163.8	57.0	21.2	10.88548542	24.34624462	12.0068139	24.3656592	12.52772068	23.20906176
C072	M	76	158.5	53.3	21.2	15.22890094	22.55333988	16.58142669	22.58978242	17.43874506	21.2169031
C073	M	77	166.2	58.2	21.1	13.16304891	23.98953832	13.01915129	24.05154684	13.89228935	22.28374653
C074	M	86	157.7	52.3	21	13.79221581	23.82625418	14.60707519	23.80274123	15.71584627	21.81023682
C076	F	53	161.0	54.0	20.8	21.0488304	22.29733613	20.53096538	22.14927231	20.88046872	21.68957058
C077	F	59	160.0	53.0	20.7	19.62294221	21.50501812	19.32018689	21.29600228	20.07385393	20.25383661
C078	F	45	157.1	50.1	20.3	19.95929664	23.34290626	19.54257588	23.27419949	20.0673222	22.55258398
C079	F	53	157.0	50.0	20.3	12.26973907	20.99730915	13.04512033	21.19904036	13.13763979	21.00657426
C080	M	83	172.0	60.0	20.3	19.85631073	24.82660893	19.61194799	24.88035038	20.46973435	23.71452292
C081	F	58	152.6	47.2	20.3	9.498293608	21.36961979	9.520944766	21.73887715	9.81661886	20.90600155
C082	F	53	153.3	47.6	20.3	15.05830469	22.03498877	16.09810108	22.09055053	16.62781774	21.20884311
C083	F	43	156.7	49.7	20.2	9.883508415	21.3587922	10.09732685	21.57797023	10.23621266	21.20593298
C084	F	38	152.5	46.8	20.1	19.15681877	22.25175378	18.6557169	22.24911957	19.51830794	21.0181546
C085	M	71	172.3	59.5	20	9.275730031	24.30993326	9.85225589	24.41999567	10.46180749	22.78514218
C086	M	79	167.4	55.6	19.8	12.29639572	23.06298689	12.06094533	23.08559779	12.64851344	21.7901747
C087	F	66	155.4	47.9	19.8	14.60807761	20.94513145	15.96978742	20.96722723	16.7935152	19.59753703
C088	F	65	156.0	48.0	19.7	16.21845154	20.80972764	16.69985819	21.00744079	16.94069973	20.61748946
C089	M	76	163.4	52.5	19.7	8.440405819	23.66822658	8.977300088	23.7189812	9.638935961	21.78235902
C090	F	54	156.8	48.2	19.6	8.89772164	18.94633981	9.229837822	19.13135874	9.490948287	18.37162199
C091	M	18	175.0	60.0	19.6	12.23852304	22.67796104	11.56809146	22.74939326	12.19469478	21.31281002
C092	F	73	151.6	45.0	19.6	10.57357605	21.58784276	12.2774836	21.54147046	12.4937266	21.06598128
C093	F	31	152.6	45.2	19.4	9.111591587	21.08727368	9.062453364	21.05373486	9.487569001	19.80527822
C094	F	64	153.0	44.8	19.1	8.732545605	19.70772429	9.323180405	19.76253252	9.780387487	18.45871691
C095	F	46	168.0	53.8	19.1	11.90738876	21.41193333	11.55122049	21.63008908	11.98003476	20.63741429
C096	F	61	154.0	45.1	19	7.137702448	21.28722275	7.667987169	21.32881696	8.000031519	20.17434917
C097	F	69	159.0	48.0	19	19.50097057	21.65695443	19.10208942	21.60517913	19.65881499	20.82280964
C098	F	19	147.9	41.2	18.8	9.313045649	19.13838416	8.741881283	19.33593729	8.736927979	19.35137269
C099	F	45	162.8	49.9	18.8	11.70798464	23.31180097	11.10559295	23.47967482	10.64215966	24.64051751
C100	M	34	167.7	52.6	18.7	9.962971532	19.67656899	10.34173911	19.72425893	10.93278877	18.21066077
C101	F	84	159.0	47.0	18.6	8.902169424	20.32561119	9.518544102	20.47656689	9.857494917	19.52365935

C102	F	34	163.2	49.5	18.6	8.91059432	20.19645536	8.634800206	20.23312397	8.775595957	19.79264504
C103	M	68	163.7	49.8	18.6	11.38163722	21.52584109	10.72456581	21.60369157	11.45594125	19.80705121
C104	M	76	164.2	50.0	18.5	19.89595605	22.98711038	19.62748842	22.93560979	20.8533899	21.28565049
T001	M	51	169.5	110.0	38.3	10.34626634	32.82539666	10.31833282	32.86221487	9.144479208	36.15126548
T002	F	64	158.2	79.4	31.7	13.1528262	31.37357255	13.41728382	31.27328777	11.19424711	36.20648018
T003	F	70	150.8	69.0	30.3	17.98335121	28.18647862	17.12392387	28.15913642	16.18260824	29.69890799
T004	M	49	183.8	100.6	29.8	28.81951359	32.05674078	28.8770975	31.98578542	26.64399665	34.17767891
T005	M	51	168.4	82.9	29.2	13.7692051	30.80609639	14.03094517	30.73829567	12.2323108	34.47431791
T006	M	72	168.7	81.2	28.5	11.85809058	28.51956136	12.0828444	28.67975808	10.51332832	32.46911537
T007	F	40	157.8	69.7	28	8.859322654	27.15262196	8.766515764	27.26359512	8.656934493	27.60615988
T009	M	72	157.0	67.7	27.5	21.428606	29.23126401	20.51152149	29.0676407	18.38783911	32.04419568
T010	M	65	181.6	90.4	27.4	18.27053301	29.63554351	17.59248332	29.82848485	16.20736855	32.06179494
T011	M	61	173.0	82.0	27.4	25.86050065	28.60752226	26.14345644	28.46291125	24.95301435	29.73211322
T012	M	83	170.0	79.0	27.3	8.460873758	25.13667732	8.654689028	25.22821134	7.965175339	27.48920605
T013	M	71	180.0	87.6	27	17.58179288	28.44163399	16.89261438	28.31921511	15.21389293	31.16968582
T014	F	48	163.0	71.1	26.8	8.131745423	27.63220992	8.157752958	27.79107999	7.87838954	28.74004094
T015	M	53	172.2	79.0	26.6	14.0699148	29.58127766	14.46934719	29.40757274	13.69174701	30.91193356
T016	M	46	184.5	90.0	26.4	10.7983396	27.99336813	11.07324859	27.94285381	10.22245408	30.12005569
T017	F	80	151.0	60.1	26.4	15.02497871	25.92987756	14.01589897	25.91828219	13.07797001	27.804567
T018	M	71	172.9	77.8	26	22.44083209	26.17944016	22.70792039	26.39855225	20.9928401	28.53726944
T019	M	73	166.6	71.9	25.9	11.41434977	26.76901728	11.94850613	26.95929157	11.29037735	28.50222272
T020	M	74	162.2	67.8	25.8	15.50326832	26.89917012	14.8394865	26.75604294	14.09109996	28.16533306
T021	F	70	146.2	54.8	25.6	16.33423711	24.62491815	16.47404349	24.81147987	15.47008256	26.52387361
T022	M	71	169.8	73.5	25.5	11.61652046	27.60160797	11.68416173	27.6937743	10.57726875	30.40425069
T023	M	64	170.5	73.8	25.4	11.4790178	28.14668362	11.7874212	28.10223502	10.93371881	30.14969607
T024	F	63	164.3	68.5	25.4	8.798151	26.77096613	8.853868368	26.82389314	8.477643187	28.00642928
T027	M	83	160.6	64.4	25	12.36684556	26.93481589	12.18730615	27.07140815	10.90286554	30.10439273
T029	M	84	168.5	70.2	24.7	11.08482004	25.75846726	10.77444937	26.08564912	9.896058036	28.4016211
T031	M	67	166.0	67.4	24.5	15.19144949	26.25299131	14.37617365	26.31658509	13.6798593	27.66866716
T032	F	74	152.2	56.6	24.4	8.271551726	25.30675606	8.24797561	25.55205583	7.880847839	26.79206343
T034	M	82	156.0	59.0	24.2	10.43261421	25.04751232	10.79556795	25.11897059	9.923014846	27.41418654
T035	M	83	164.7	65.6	24.2	13.25529707	25.53378704	12.47621511	25.53015198	11.8023749	27.04224944
T036	M	80	147.5	52.5	24.1	14.76759231	24.88475484	13.53644964	25.14559476	11.70946621	29.09414909
T037	M	76	164.5	64.9	24	13.51408833	26.25939662	12.84878245	26.26783334	11.85383935	28.46278507
T038	F	85	140.0	47.0	24	21.73320314	23.64342683	21.64561583	23.93164352	20.86716155	24.92910688
T039	M	77	177.4	75.1	23.9	13.08714572	28.73143587	13.06023613	28.90429703	11.57630513	32.18898999
T040	M	80	162.5	63.0	23.9	7.796588578	26.0104723	7.996615131	25.96071741	7.389228801	28.11203349
T042	F	86	141.6	47.7	23.8	11.29435008	23.78767672	10.72980336	24.03085489	10.53925346	24.51884037
T043	M	67	166.0	65.5	23.8	12.01411659	25.21296234	11.44464499	25.2214222	10.9035305	26.5404889
T044	M	72	173.9	71.7	23.7	8.430990597	26.58834425	8.594898896	26.54719131	7.942424967	28.69728908
T045	F	78	142.4	48.0	23.7	9.504746817	21.87355293	9.605071119	21.97211728	9.095829533	23.45567174
T046	M	69	165.0	63.4	23.3	11.03559619	25.80683828	11.36248882	25.83341339	10.29035205	28.53255318
T047	M	71	171.0	68.0	23.3	8.106419413	25.54679765	8.38436347	25.48563701	7.698486825	27.80987857
T048	M	66	174.4	70.5	23.2	11.17422769	25.67009974	11.52634691	25.71719027	10.95527557	27.10104633
T049	M	51	169.3	66.4	23.2	15.8235188	26.92923785	15.2332486	26.79587803	14.18521327	28.73709716
T050	M	87	161.1	60.0	23.1	7.852756801	23.98146943	7.867240011	24.07063928	7.520878362	25.29681415
T051	M	67	162.0	60.2	22.9	18.38510378	25.11499633	18.85312181	25.09569298	17.23100974	27.54584017
T052	M	59	166.8	63.8	22.9	7.510104002	24.20429327	7.635053792	24.40540764	7.160685907	26.1522843
T053	M	82	163.1	61.0	22.9	11.23988327	26.59398969	11.54832181	26.536332	9.870310283	30.81224518
T054	M	50	177.5	72.0	22.9	14.17227369	25.44954168	13.23633124	25.4585504	12.22880574	27.61466365
T055	M	90	160.0	58.5	22.9	10.22447999	25.24002546	10.34691275	25.4833476	9.525826153	27.73506209
T056	M	56	175.0	69.4	22.7	11.32401263	28.654118	11.69703039	28.55476574	10.84856676	30.60550958
T057	M	60	163.0	60.2	22.7	8.439966725	23.91776924	8.208038238	24.41049835	7.729293197	26.04714203
T058	M	74	166.0	62.4	22.6	14.00484469	24.75509903	14.09352421	24.60398452	13.64218736	25.49039442
T059	F	38	160.0	57.8	22.6	12.6176269	23.53535519	12.06908285	23.77463505	12.58007555	22.64533435
T060	M	71	173.2	67.7	22.6	21.07068304	25.81714497	21.74672681	25.63844833	20.87691064	26.75010848
T061	F	85	158.8	56.9	22.6	22.81689872	21.5752514	23.0881233	21.98040883	23.22961634	21.81402013
T062	M	52	166.2	62.2	22.5	23.5085733	24.51045539	23.28302077	24.84409396	22.03720676	26.34172671
T063	M	70	167.6	63.0	22.4	12.65556136	25.67862356	12.08245473	25.76270403	11.46997691	27.17943478
T064	M	68	175.0	68.6	22.4	8.271019987	26.74425883	8.424614683	26.73558394	7.6803782	29.25438939
T065	M	64	170.7	65.2	22.4	15.42477877	25.40698782	14.45194017	25.63413679	14.11539226	26.27583685
T066	M	67	166.0	61.6	22.4	17.96821256	24.56435534	18.11143813	24.85784796	17.13252294	26.37108459

T067	M	72	170.2	64.7	22.3	15.51369423	26.33224339	14.83698243	26.2467824	13.68578051	28.44631639
T068	M	56	181.2	73.2	22.3	11.36581155	26.73132207	11.74263854	26.76562549	10.93122542	28.71563513
T069	M	71	168.5	63.0	22.2	7.718117656	27.12382074	7.954095111	27.10848798	7.228744918	29.71260338
T070	M	77	159.0	56.0	22.2	9.653280863	23.18007275	9.801866154	23.32056555	9.473930456	24.24729516
T071	M	50	171.4	64.7	22	23.16467514	24.61779059	23.71788106	24.68596807	22.51167338	26.10743165
T072	F	80	143.0	45.0	22	10.46118548	23.17187227	10.1671029	23.05741145	10.38798452	22.47209362
T073	M	79	161.4	57.3	22	13.04147868	24.03099326	12.38314019	24.10284029	11.70052973	25.64703258
T074	F	82	155.3	52.9	21.9	11.22967625	23.18690997	10.65160264	23.44063045	10.02863118	25.08189619
T075	M	39	167.1	61.0	21.8	23.72221713	23.98238469	23.81899167	24.34582862	23.39057737	24.84011725
T076	M	89	169.4	62.0	21.6	11.44823174	24.93146144	11.3691315	25.22657273	10.79241485	26.6443084
T077	M	77	152.4	50.0	21.5	7.657126284	24.16160856	7.836086743	24.17869488	7.382217086	25.8036018
T078	F	79	145.0	45.0	21.4	7.271423588	22.31515155	7.262493678	22.38432871	7.260411257	22.39213869
T079	M	74	172.6	63.7	21.4	12.94955654	24.48897776	12.32743905	24.55977575	10.96784926	27.74227579
T080	M	32	158.5	53.1	21.1	22.12238939	23.92517514	22.36608893	24.17720133	21.78959272	24.8883646
T083	M	82	166.2	58.0	21	11.11108498	23.79413121	10.67266135	23.78441917	9.828077099	26.02961451
T084	F	81	156.2	51.2	21	11.72855897	23.41258504	11.29147834	23.4085075	11.25064013	23.50718251
T085	M	90	168.0	59.0	20.9	8.466521322	24.69524411	8.961474814	24.64635664	8.697151513	25.46170978
T086	F	84	160.1	52.8	20.6	10.8915805	23.95706608	10.5968525	24.33147593	10.30733849	25.08587087
T087	M	72	171.2	60.2	20.5	8.516153878	26.04129235	8.410325065	26.19222339	7.491454654	29.34307086
T088	M	67	161.8	53.7	20.5	7.760315824	24.75810899	7.944715238	24.68990648	7.179386964	27.44847667
T089	F	79	143.3	41.4	20.2	10.0682314	21.80094321	9.7821012	21.89337609	9.906509805	21.5492038
T090	F	59	160.5	51.7	20.1	11.69607684	22.96627936	11.06452618	23.19426863	11.38843091	22.40847475
T091	M	84	169.9	57.7	20	11.1988021	23.33550819	12.35242122	23.42057458	11.76564369	24.74598841
T092	F	76	154.6	47.5	19.9	10.0701056	22.23488919	10.41994688	22.21768437	9.928334267	23.53386223
T093	M	76	162.0	52.0	19.8	10.53056616	23.17156129	10.13216256	23.15116376	9.875769137	23.84917631
T094	M	64	166.5	54.9	19.8	15.31001997	24.60006511	14.76327659	24.50348506	13.68769585	26.5635822
T095	M	81	170.0	57.2	19.8	10.1601341	25.26616641	10.28811309	25.48512356	9.386721903	27.98225336
T096	M	67	169.8	57.0	19.8	7.537356825	23.9289058	7.662999703	24.08933856	7.331495203	25.29371745
T097	M	70	164.0	53.0	19.7	11.37715835	22.6785194	11.48204181	22.9527284	10.75714062	24.72875166
T098	F	68	156.9	48.3	19.6	7.847568409	21.86425013	7.905018034	22.0763891	7.502844234	23.49840621
T099	M	69	171.0	57.0	19.5	11.93194023	23.50604228	11.2596204	23.48545332	10.72371309	24.81351107
T100	M	70	171.5	57.0	19.4	18.8206584	24.09958203	18.87695061	24.23990116	17.27027775	26.66245522
T101	M	73	164.0	52.0	19.3	7.703788442	24.01973072	7.700122675	24.17429593	7.225653635	25.90631263
T102	M	64	171.0	56.0	19.2	7.8369534	22.68723459	7.702120856	23.01052354	6.833401464	26.26965242
T103	F	80	154.0	45.4	19.1	7.316118757	21.17726137	7.440893247	21.23050468	7.560379507	20.79666045
T104	M	82	161.0	49.4	19.1	18.85957122	23.56858364	19.29665239	23.64103478	17.40277939	26.4543102
T105	M	79	164.3	51.2	19	11.40526801	22.05606888	10.66719659	22.17199853	9.952965598	24.05936033
T106	M	55	169.0	53.8	18.8	11.54639181	23.34959774	11.00779077	23.33427313	9.678069563	26.84034685
T107	M	80	156.7	45.9	18.7	12.33316117	21.41489619	11.24557473	21.55935047	10.84808749	22.53937539
